# One mental health problem influencing the risk for another, within individuals and between siblings. Commentary on Allegrini et al. (2022)

**DOI:** 10.1002/jcv2.12102

**Published:** 2022-08-24

**Authors:** Christel M. Middeldorp

**Affiliations:** ^1^ Child Health Research Centre The University of Queensland Brisbane Australia; ^2^ Child and Youth Mental Health Service Children's Health Queensland Hospital and Health Service Brisbane Australia

Co‐occurrence of mental health symptoms, either at the same time or across development, is a major area of research. It is well known that comorbidity is associated with poorer outcomes and continuation of childhood symptoms into adulthood (Costello & Maughan, 2015). Better insight into mechanisms underlying the development of comorbidity could provide leads for interventions to prevent the continuation of mental disorders.

So far, twin studies have mainly focused on genetic and environmental factors influencing the continuation of symptoms or the development of comorbid symptoms over time in childhood (Kuja‐Halkola et al., 2015; Wertz et al., 2015). Allegrini et al. (2022) have taken a rather novel approach in exploring mechanisms underlying co‐occurrence of symptoms over time. Focusing on social, internalising, attention deficit hyperactivity disorder (ADHD) and externalising symptoms, they not only included between person processes in their longitudinal model, like in the before mentioned studies, but also within person processes to explain the co‐occurrence of symptoms. In between person processes comprise of factors that influence two or more psychopathologies, whereas in within person processes one type of psychopathology influences the other. A genetically informed study applying Mendelian Randomisation analyses suggested that childhood maltreatment is causally associated with depression (unidirectional) as well as with (ADHD) and schizophrenia (bidirectional) (Warrier et al., 2021), that is, childhood maltreatment could be part of the between person effects explaining the comorbidity between these disorders. An example of a within person process is that someone with social anxiety doesn't have friends and then gets depressed because of that. Knowledge on which of these two processes explains the co‐occurrence can aid in choosing the right treatment strategy. While in the first example all disorders need to be addressed in treatment, in the second example a successful treatment of the social anxiety could be sufficient as it will lead to a decrease of the depressive symptoms.

Allegrini et al. tested the between and within person processes in a random intercept cross‐lagged panel model (RI‐CLPM) using data collected at age 7, 9/10 and 12 in the Twin Early Development Study (TEDS) (N varies between 5828 and 7758) and the Netherlands Twin Register (NTR) (N varies between 8748 and 12,710). In addition, they extended their model to family data allowing to explore the existence of reciprocal directional influences between siblings (within family effects) besides between family effects, such as genetic effects, to explain resemblance in psychopathology between siblings. The sibling analyses are presented as supplementary analyses, I think, because they are not mentioned in the preregistered protocol at the open science forum, which shows the scientific rigour of the authors.

The first analyses where they tested the RI‐CLPM using data from one individual per family showed that the majority of the variance in social, internalizing, ADHD and externalising problems was explained by between person processes, ranging from 42% to 57% in both cohorts. In addition, significant within person effects were detected, explaining between 4% and 11% of the variance. In this commentary, I will only focus on the within person processes that were found in both cohorts as these results seem more robust. These were effects from social problems to internalising problems and from externalising problems to attention and internalising problems for the time lag age 7–9/10 and from internalising problems to social problems for the time lag age 9/10 to age 12. The authors emphasize that these directed influences do not need to be causal influences as there still may be confounding factors at play. They for example, mention that developmental genetic changes, that is, new genetic effects coming into play at a later age, probably would be pushed into the within person processes. If in future analyses, the reported effects were found to be causal, this would underline the importance of intervention in the early stage of a disorder, as that would mean that early intervention, besides minimising the impact on, for example, school and friendships of an ongoing mental disorder, could prevent the development of future symptoms.

Although supplementary, I was fascinated by the results of the sibling analyses that indicated significant within‐family reciprocal relationships between siblings over time. Higher externalizing problems in one sibling at age 7 predicted higher internalising problems for their sibling at age 9/10 in both cohorts. This suggests that resemblance between siblings is not only explained by shared familial, mainly genetic, effects, but also by symptoms of one sibling influencing the symptoms of the other. So far, most literature has focused on how parental and offspring mental health problems influence each other and not so much on siblings. The parent‐offspring studies have shown parent to child (Ahmadzadeh et al., 2021; Speyer et al., 2022) and child to parent effects (Speyer et al., 2022). Research in a clinical sample suggested that outcomes for children whose parents report mental health problems are worse than for children whose parents don't report these problems and that this is partly explained by ongoing associations with parental mental health problems (Wesseldijk et al., 2018). The results from Allegrini et al. indicate that we should also be aware of siblings influencing each other. Again, the within family effects are smaller than the between family effects, just as the within person effects are smaller than the between person processes. Still as these effects may be easier to target in treatment, if the results are replicated, the recommendation to screen parents for mental health problems when children are referred to a psychiatric clinic may need to be extended to the whole family.

As the authors have also pointed out in their paper, there are multiple ways to follow up on this research. Firstly, it would be good if their results could be replicated as well as extended to other ages. There are multiple birth and childhood (twin) cohorts with similar data that, like TEDS and NTR, also include older ages. Moreover, many of those also have genetic data available providing the opportunity to add polygenic risk scores to the analyses (Middeldorp et al., 2019). Polygenic risk scores reflect an individual's genetic vulnerability to a trait of interest. So far, polygenic risk scores have been used to explore whether genetic variants for different psychopathologies overlap. Overlap can indicate genuine pleiotropy where genetic variants increase the risk for two mental disorders but can also indicate that one mental disorder increases the risk for another disorder. By including these scores in a RI‐CLPM it can be tested whether a polygenic risk score is mainly associated with one psychopathology that increases the risk for another or whether the polygenic risk is associated with both of them. Finally, as suggested by Allegrini et al., including polygenic risk scores to a model including family data allows for testing of direct and indirect genetic effects.

To conclude, the results of Allegrini et al. suggest that intervening in an early stage of a mental disorder and taking a family based approach could aid in preventing the development of additional problems either in the individual or in their family members. Replication and extension of their results is necessary to be able to make more specific recommendations, also keeping in mind that between person and between family processes do explain more of the individual differences.

## AUTHOR CONTRIBUTIONS

**Christel M. Middeldorp**: Writing – original draft.

## CONFLICTS OF INTEREST

Christel M Middeldorp serves on the JCPP *Advances* Editorial Advisory Board.
